# Breastfeeding Media Coverage and Beliefs During the COVID-19 Pandemic in Mexico: Implications for Breastfeeding Equity.

**DOI:** 10.21203/rs.3.rs-528093/v1

**Published:** 2021-05-19

**Authors:** Mireya Vilar-Compte, Pablo Gaitán-Rossi, Elizabeth C. Rhodes, Valeria Cruz-Villaba, R. Pérez-Escamilla

**Affiliations:** Universidad Iberoamericana; Yale University School of Public Health; Universidad Iberoamericana; Yale University School of Public Health

**Keywords:** breastfeeding, COVID-19, infant feeding, inequities, media analysis

## Abstract

**Background:**

Breastfeeding offers short- and long- term health benefits to mothers and children and constitutes a priority for public health. Evidence shows that SARS-CoV-2 is not likely to be transmitted via breastmilk. Moreover, antibodies against SARS-CoV-2 are presumably contained in breastmilk of mothers with history of COVID-19 infection or vaccination. Direct breastfeeding is the preferred infant feeding option during the pandemic, but conflicting practices have been adopted, which could widen existing disparities in breastfeeding. This study aims to describe how was information about breastfeeding communicated in Mexican media during the pandemic and assess Mexican adults’ beliefs regarding breastfeeding among mothers infected with COVID-19.

**Methods:**

A retrospective content analysis of media coverage on breastfeeding in Mexico between March 1 and September 24, 2020, excluding advertisements, was done. For the content analysis, both a sentiment analysis and an analysis based on strengths, weaknesses, opportunities and threats for breastfeeding promotion were performed. Also, we incorporated a descriptive analysis from the July 2020 wave of the ENCOVID-19 survey, which included questions on beliefs about breastfeeding. This information was stratified by gender, age, and socioeconomic status.

**Results:**

1014 publications on breastfeeding were identified in internet, newspapers, TV, and magazines. Most information was published during World Breastfeeding Week, celebrated in August. Based on the sentiment analysis, 57.2% of all information was classified as positive, and based on the SWOT analysis, most information was classified either as strengths or opportunities for breastfeeding promotion. However, the ENCOVID-19 data showed that 67.3% of people living in households with children under 3 years of age believe that mothers with COVID-19 should not breastfeed, and 19.8% stated that they simply didn’t know. These beliefs showed differences both by gender and by socioeconomic status.

**Conclusions:**

While the Mexican government endorsed the recommendations on breastfeeding during the COVID-19 pandemic, communication of those messages was sporadic, inconstant and unequal across types of media. Moreover, there were also negative messages for breastfeeding circulating on the media. There continues to be a widespread notion that mothers with COVID-19 should not breastfeed and, due to differences on beliefs by socioeconomic status, health inequities could be exacerbated.

## Background

The protection, promotion, and support of breastfeeding is a priority for public health, since breastfeeding offers mothers and children a constellation of short- and long-term health benefits (1, 2). Evidence has consistently shown that the severe acute respiratory syndrome coronavirus 2 (SARS-CoV-2) is not likely to be transmitted via breastmilk (i.e. vertical transmission) (3–7). In addition, COVID-19 appears to be less prevalent and, generally, less severe in infants (8, 9), and there is evidence that breastmilk from mothers with a history of COVID-19 infection contains specific IgA with an activity against SARS-CoV-2 (9–13). Furthermore, prospective cohort studies have found that anti-SARS-CoV-2 IgA and IgG generated by anti-COVID-19 mRNA-based vaccines administered to lactating and pregnant mothers are transferred to their babies via breastmilk (14, 15) and umbilical cord blood (16, 17), while COVID-19 mRNA does not (18, 19). Moreover, evidence suggests that vaccine-induced immune responses are even greater than natural infection-induced immunity (17), due to an IgG-dominant response (20). In general terms, breastmilk protects babies through anti-microbial and anti-inflammatory factors that promote the development of the immune system (21, 22). Accounting for this evidence, as well as the susceptibility of newborns to person-to-person spread of COVID-19 through contact with mothers and caregivers (i.e., horizontal transmission) (9), the World Health Organization (WHO) recommends that women with COVID-19 should breastfeed their babies (21, 23) and that direct breastfeeding should be supported as the preferred infant feeding option during the pandemic (i.e. feeding directly from the breast). WHO also recommends that breastfeeding women to be vaccinated against COVID-19.

Protecting, promoting and supporting breastfeeding even among infected mother-infant dyads is important given the numerous health benefits of breastfeeding (24) and COVID-19-affected or -suspected mothers should be informed about the importance of continued direct breastfeeding. During the birth hospitalization period, mother-infant dyads should be cared for together, including skin-to-skin contact and room sharing, which is critical for helping mothers establish and continue breastfeeding (8). Not doing so increases neonatal morbidity and mortality (25).

Despite the WHO’s strong emphasis on promoting breastfeeding and keeping the mother-infant dyad together during the COVID-19 pandemic, some governments, professional organizations, and hospitals have adopted conflicting practices (26). During the early stages of the pandemic, for example, they recommended that infected mothers be separated from their infants after birth to reduce the risk of infant COVID-19 infection. Such recommendations generated uncertainties for new parents (8, 27). Sola et. al. (28) analyzed evidence from 7 countries of the Ibero American Society of Neonatology (Argentina, Colombia, Ecuador, Equatorial Guinea, Honduras, Peru and Dominican Republic) from March to May 2020, to evaluate how has the pandemic impacted pregnant and breastfeeding women and newborns in Latin America. Findings showed that lack of breastfeeding support and mother-infant dyad separation among COVID-19 positive women were common during the pandemic. For example, only 24% of infected mothers were allowed to breastfeed after birth and only 13% expressed milk while in the hospital; as a result, 63% of infants born to infected mothers were fed with breastmilk substitutes (BMS). Moreover, 76% of the dyads were separated at birth and 95% of mothers were left unaccompanied during delivery and the postpartum birth hospitalization period, which could possibly have disrupted mother-child bonding (28).

Diverse socio-cultural beliefs about breastfeeding, uncertainty and social anxiety (9), and the marketing strategies from the BMS industry might have also prompted healthcare providers and mothers to not start or discontinue breastfeeding during the pandemic. Furthermore, the initial United Kingdom (UK) vaccination policy that denied access to vaccines to breastfeeding women due to safety concerns, because they were not included in the vaccine trials, very likely led providers and mothers to decide to not breastfeed their infants (29), even if it was later reversed (30). Indeed, it has been noted that federal and local governments and certain COVID-19 vaccination centers in several countries, such as Spain (31), Canada (32), United States of America (USA) (33), or the UK (34), are handing out consent forms or publishing factsheets stating that breastfeeding is contraindicated or that women should contact their health care providers to further discuss the safety of the vaccine during breastfeeding, even though now there is evidence that the vaccine is not harmful for the baby nor the breastfeeding mother (31) and that actually is beneficial to both (17). In fact, evidence states that the vaccine-induced immune responses in pregnant and lactating women are equivalent to the vaccine-induced immune response in non-pregnant women (17). Nonetheless, these messages may negatively affect mothers’ decisions regarding both vaccination – for example, they may decide to not receive the COVID-19 vaccine or voluntary postpone vaccination – and infant feeding decisions, such as breastfeeding termination or non-disclosure of breastfeeding status at vaccination centers (31).

Separation of mothers from their infants during the birth hospitalization and suggesting mothers not to initiate or discontinue breastfeeding to reduce the risk of COVID-19 in infants may widen existing disparities in breastfeeding. Prior to the COVID-19 pandemic there were enormous differences in access to quality maternity services and infant feeding information by socioeconomic status (8). Furthermore, socio-economically marginalized groups are least able to follow lockdowns and social distancing guidelines to reduce the spread of COVID-19, due to the nature of their jobs (35). These groups are, therefore, least able to minimize their viral exposure. They might also be more likely to suffer the adverse consequences of mother-infant separation during the birth hospitalization, and in turn, to face barriers to breastfeeding (36). Indeed, vulnerable groups have also suffered disproportionately from the adverse economic consequences of the pandemic (35). Hence, the protection afforded to infants through breastfeeding in the context of COVID-19 – as a public health emergency – is of increased importance to mitigate household food insecurity, due to the high cost of BMS and the stress this can imply to families’ food expenditure (8). Breastmilk provides the cleanest, safest and most affordable form of infant and young child nutrition during crises, and it is the normative standard for infant nutrition (3). Hence, breastfeeding should be considered as a fundamental protective and health promotion measure for infants during the pandemic (22).

The aims of this study were to (a) describe how the support from the Mexican government in adhering to the WHO recommendations on breastfeeding during COVID-19 was communicated in media outlets, (b) to assess the beliefs among Mexican adults regarding breastfeeding among mothers infected with COVID-19, and (c) address if there are differences in views by socioeconomic status, with the goal of understanding whether socio-economically disadvantaged families might be more likely to hold views that do not align with current scientific recommendations regarding breastfeeding.

This research is needed because Mexico has been severely affected by the COVID-19 pandemic; it has the third highest number of COVID-19 related deaths in the world (37). The public health emergency in Mexico has had sustained negative effects on household income, employment, food insecurity, and mental health since the beginning of the pandemic in March 2020 (38, 39). Prior to the pandemic, there had been a steady increase in breastfeeding rates in Mexico. For example, the exclusive breastfeeding rate among infants under six months improved from 14.4% in 2012 to 28.3% in 2018 (40, 41). Due to the COVID-19 pandemic, however, these improvements may slow down and widen breastfeeding inequities if actions to promote and protect breastfeeding during this public health emergency are not taken.

## Methods

The study used two complementary methodological approaches: (a) a retrospective content analysis of media coverage on breastfeeding in print and online media sources in Mexico between March 1, 2020 and September 24, 2020, and (b) a descriptive analysis based on nationally representative data from a phone-based survey, the Survey on COVID-19 Effects on Wellness in Mexican Households (ENCOVID-19, for its acronym in Spanish).

### Media analysis

The analysis included any newspaper, radio, and television coverage of breastfeeding. The media search was conducted by *Eficiencia informativa,* a company that specializes in media monitoring and content analysis. It was done using a combination of the following key words in Spanish: breastfeeding, maternity leave, Becoming Breastfeeding Friendly, and Permanent Interinstitutional Support Group for Breastfeeding (GILPALM, for its acronym in Spanish, a group led by the Ministry of Health).

The inclusion criteria for the media search were: (a) date of publication between March 1, 2020 and September 24, 2020; (b) includes information on breastfeeding; (c) published and disseminated in Mexico, and (d) not an advertisement.

The information identified in the media search was organized both by media platform and by date, in order to identify the frequency of documents or programs mentioning breastfeeding per month; the unit of analysis in this sense was the document or program (i.e. if a document mentioned more than once breastfeeding it will still be counted as a single unit). For the content analysis, those documents or programs that assessed breastfeeding in relationship to the COVID-19 pandemic were differentiated from those that only assessed breastfeeding in order to analyze how breastfeeding information in the context of the COVID-19 pandemic was reported in traditional media. A sentiment analysis was performed, and each piece of information was classified as positive, negative, or neutral with regards to its potential impact on breastfeeding promotion; examples of such classification can be found in Table 1.

Additionally, an analysis was conducted to identify strengths, weaknesses, opportunities, and threats (SWOT) for breastfeeding promotion, with the aim of generating information that can be used to identify actions for improving breastfeeding promotion in Mexico during the COVID-19 pandemic (42), but this time focusing on the available information about breastfeeding related to the COVID-19 pandemic. The operational definitions for each category of the SWOT analysis and illustrative examples are presented in Table 2.

### Descriptive analysis of ENCOVID-19

The ENCOVID-19 is a monthly telephone survey designed to document the consequences of the COVID-19 pandemic in Mexico. With a repeated cross-sectional design, starting in April 2020, the ENCOVID-19 is administered monthly to a nationally representative sample of individuals 18 years and older who have a mobile phone. Since national mobile phone coverage in Mexico was 89.4% in 2019, including 74% in rural areas (43), post-stratification sampling weights were used to correct minor deviations from Mexico’s demographic structure.

The ENCOVID-19 survey included two questions on breastfeeding in the July 2020 wave. The sample included 1,584 individuals and was representative of households with children under 18 years of age (57.8%). Amongst them, 234 (17.7%) people lived in a household with a child under 3 years old. Participants were asked to respond to the following two questions: “From what you know, if a mother has COVID-19, should she breastfeed?” Response options were Yes, No and Don’t know. If they expressed a negative answer, the follow-up question was: “What is the reason not to breastfeed?”. Response options were i) “The virus is transmitted through breastmilk”; ii) “My physician recommended not to”; and iii) “The mother should be isolated”.

In addition to collecting data on demographic characteristics (i.e., sex and age in years), the ENCOVID-19 collects data for calculating the Mexican Association of Market and Opinion Intelligence Agencies (AMAI, for its acronym in Spanish) index (44), an assets-based household socioeconomic index with six indicators, including: (i) education level of the head of household; (ii) number of bathrooms; (iii) number of cars or vans; (iv) having an Internet connection; (v) number of household members 14 years or older who are working, and (vi) number of bedrooms.

Descriptive analyses were conducted to generate frequencies and proportions of responses to each question on breastfeeding by gender, age, and socioeconomic status.

## Results

### Media analysis

**Figure 1:**
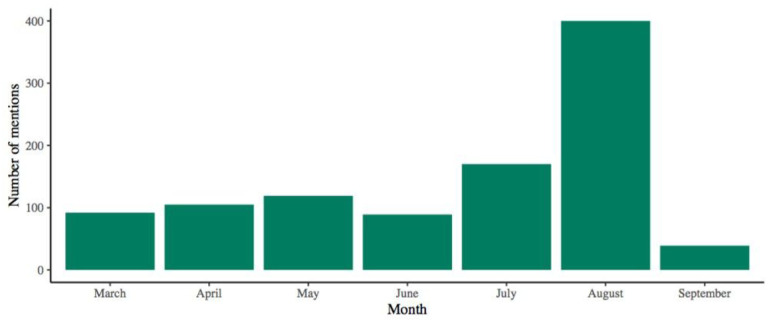
Total breastfeeding mentions in media outlets by month from September to March 2020

A total of 1014 mentions of breastfeeding were identified; classification by type of media is shown in Table 3. During August 2020, when World Breastfeeding Week was celebrated in Mexico, these mentions spiked, reaching 400 during the whole month (Fig. 1).

**Figure 2:**
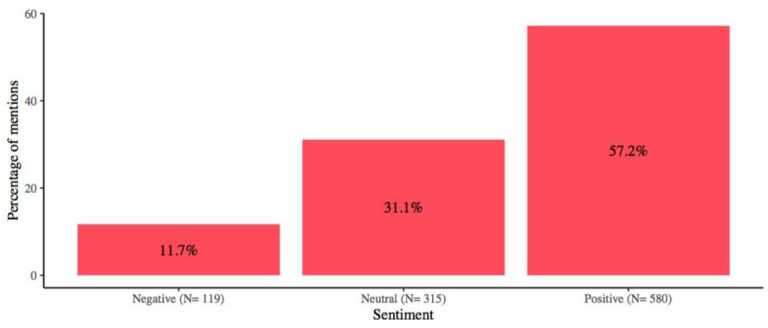
Breastfeeding mentions’ sentiment analysis

More than half of all information was classified as positive for breastfeeding promotion (Fig. 2), and in all media outlets, positive information was higher than negative and neutral information combined, except for information published in magazines, where most of the information on breastfeeding was classified as neutral.

The most prevalent negative theme was the increase of marketing of breast milk substitutes during the pandemic, accounting for 67 (6.6%) of total mentions of breastfeeding. Six (0.6%) mentions stated that COVID-19 was detected in breastmilk and 4 (0.4%) mentions referred to the influence of advice from medical doctors in women’s decisions not to breastfeed. Positive information on breastfeeding is shown in Table 4.

In accordance with the sentiment analysis, in the SWOT analysis, almost all of the information (84.6%) was labeled as positive for the promotion of breastfeeding, since 64.3% of all publications were classified as strengths and 20.3% as opportunities, whereas 11.7% of them were classified as threats and only 3.6% as weaknesses. Relevant results from the SWOT analysis are shown in Table 5.

### ENCOVID-19

**Figure 3:**
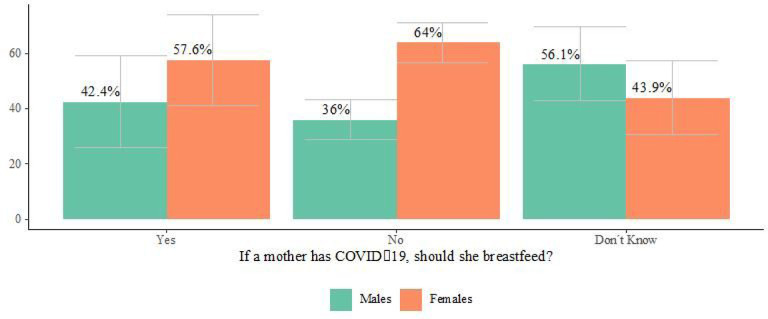
Views of breastfeeding during the COVID-19 pandemic among adults living in households with children 3 years and younger (ENCOVID-19- July 2020. N= 234) *Bars represent confidence intervals

Our analysis of ENCOVID-19 survey data found that 67.3% of people living in households with children under 3 years of age believed that mothers with COVID-19 should not breastfeed, and 19.8% reported that they did not know if mothers with COVID-19 should breastfeed. Notably, the proportion of women who thought mothers should not breastfeed was higher among women than in men, while more men reported that they simply did not know, in comparison to women (see Fig. 3). Views on breastfeeding during the COVID-19 pandemic did not differ according to age.

**Figure 4:**
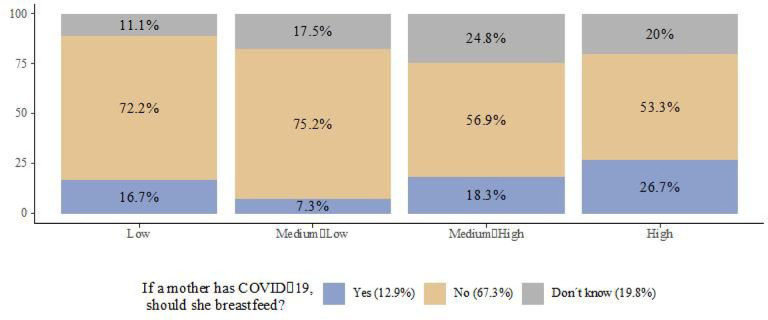
Views of breastfeeding during the COVID-19 pandemic by socioeconomic status in households with children 3 years and younger (ENCOVID-19- July 2020. N= 234)

People living in households with low and medium-low socioeconomic status had the highest proportions of respondents indicating that mothers with COVID-19 should not breastfeed, 72.2% and 75.2%, respectively (Fig. 4), but even among those households with high socioeconomic status that had the highest proportion of affirmative answers, only a minority of them considered that mothers with COVID-19 should breastfeed (26.7%). The share of people stating that they did not know the answer ranged from 11% in households with low socioeconomic status to 24.8% in households with medium-high socioeconomic status.

Most respondents who reported that women infected with COVID-19 should not breastfeed, expressed that the reason why they should not do so was because “the virus is transmitted by milk” (62.4%), followed by the belief that the “mother should be isolated” (23.7%). Only a few respondents reported that their “physician recommended not to” (1.6%), while 12.4% said it was due to “Other” reasons.

## Discussion

The COVID-19 pandemic is posing challenges to breastfeeding due to fears of potential vertical transmission and the exclusion of pregnant and breastfeeding women from vaccine trials, leading to initial recommendations to exclude this group from immunization. Evidence supports the continuation of direct breastfeeding, skin-to-skin contact, not separating the mother-infant dyad during the birth hospitalization even if mothers are infected by of SARS-CoV-2, and the inclusion of pregnant and breastfeeding women in the COVID-19 vaccination schemes. Despite such evidence it has been hard to combat the public unsubstantiated fears of infection initially divulged.

This study documents important lessons from Mexico, which might be relevant for other countries. First, as shown by our media analysis, the government and health organizations of the country backed the recommendations of the WHO to directly breastfeed infants even if mothers test positive for SARS-CoV-2. Second, despite this recognition and adherence to WHO recommendations, the media analysis revealed that the promotion of such messages was unevenly distributed by type of media outlet. This is important because women from different socioeconomic backgrounds and educational levels may be exposed to different media outlets. While almost all of the information regarding breastfeeding was distributed in digital media, access to internet is deeply unequal by type of location (i.e., urban or rural), gender, educational level, occupation (i.e., workers and students or housewives), and socioeconomic status in Mexico (45). There is a difference of 70.7 percentage points in access to internet connection between households with high and low socioeconomic status in Mexico. Regarding public television and radio, this gap is far smaller, since there is a difference of only 13.9 and 8.8 percentage points, respectively. Even if public television is the most used and evenly distributed type of media in the country, most information on breastfeeding was distributed in internet, which shows the widest distribution gap by socioeconomic status. Therefore, vulnerable households with poor access to internet have been at higher risk of no receiving such information. This study also revealed an imbalanced distribution of messages across time, as most happened during World Breastfeeding Week in August. Informing families about infant feeding choices amidst a pandemic, probably requires a more constant strategy. Third, the media analysis showed that the presence of marketing strategies and promotion of breast milk substitutes during the pandemic. This finding is consistent with observations documented in other regions during the same period that document how BMS companies are capitalizing on fear of breastfeeding during the COVID-19 pandemic to market their products (46). Such strategies to promote and distribute BMS during fragile circumstances such as earthquakes (47, 48) and tsunamis (49), have been documented internationally (50). Prior evidence highlights that governments and international organizations need to step in to regulate such donations and guard mothers and infants from unnecessary promotion and distribution of BMS that usually reaches the most vulnerable. For this reason, the World Health Assembly urges all state members to ensure evidence-based and appropriate IYCF during emergencies (51). Lastly, data from the ENCOVID-19 showed that despite the positive messages that could have been promoted by the government about breastfeeding during the pandemic, they were insufficient to change public opinion, as most respondents believed that an infant should not be breastfed if the mother is infected with COVID-19. Such beliefs were more common among lower socioeconomic groups, which could indeed lead to inequities in breastfeeding during a public health emergency. This is worrisome in light of the increased prevalence of food insecurity in Mexico during the pandemic, which has particularly affected lower socioeconomic status groups (39). Decreases in breastfeeding rates can exacerbate household food insecurity due to the high costs of BMS. Furthermore, formula feeding is associated with increased morbidities in infants and young children. Furthermore, prior research has documented increased risk of SARS-CoV-2 infection among low income households due to their type of housing, employment and transportation (52, 53). This could imply an increased risk of infection among pregnant and lactating mothers of more vulnerable groups, which in turn could be associated with inadequate initiation of breastfeeding and early weaning.

Hence, breastfeeding promotion, protection, and support strategies must be prioritized by health professionals, the government, and advocacy groups targeting pregnant women and breastfeeding mothers of vulnerable socioeconomic groups in order to provide correct information to mothers and families about breastfeeding during the pandemic. Evidence-based and consistent messaging through diverse types of media outlets, accompanied by strong breastfeeding protection, promotion and support at clinics and hospitals, are strategies that need to be implemented. Governments are facing unprecedented challenges to provide health services amidst the pandemic, infant and young child feeding information and services during the COVID-19 pandemic needs to be immediately prioritize due to the long-term and inequitable effects of not doing so.

This study is novel in that is uses a mixed-methods approach; the media analysis allowed identifying how WHO breastfeeding recommendations during the COVID-19 pandemic were promoted and divulged, while the telephone survey captured beliefs around breastfeeding during the pandemic among members of families with children under 3 years of age. Our analyses documented breastfeeding promotion and beliefs gaps and identified subgroups particularly affected, suggesting the need to prevent breastfeeding inequities affecting socio-economically vulnerable groups during a major public health emergency. This methodology can be easily and rapidly deployed in other countries. The analyses had some limitations, mainly that the media analysis excluded social media (i.e. Twitter). Second, the ENCOVID-19 sample is cross-sectional, precluding drawing causal inferences from findings’ analysis of a relatively small sample that should be expanded.

## Conclusions

The Mexican government publicly communicated the WHO recommendations on breastfeeding during the COVID-19 pandemic, and messages appeared in different media outlets. However, communication was sporadic, inconstant and unequal across different types of media. In addition, the media analysis revealed that while the government was promoting breastfeeding, there were other media messages linked to donations and promotion of BMS. The survey on breastfeeding beliefs during the pandemic among adults living in households with children under 3 years reveals the widespread notion that if a mother is infected with COVID-19 should not breastfeed. Such belief was more prevalent among socio-economically disadvantaged families, which could be at higher risk of not initiating or discontinuing breastfeeding during the pandemic. This health inequity should be addressed through targeted actions.

## Figures and Tables

**Table 1:** Examples of positive and negative sentiment classification for breastfeeding promotion

Positive information	Neutral information	Negative information
Recommendations to continue breastfeeding during the pandemic	Reports on the importance of traditional midwifery in indigenous communities during the pandemic	Reports of increase marketing of breast milk substitutes during the pandemic
Reports of no evidence that COVID-19 can be transmitted via breastmilk	Reports on decrease in Mexican women’s fertility rate	Reports of detection of COVID-19 in breastmilk
Promotion of breastfeeding continuation during social distancing and lockdown measures by the Mexican Ministry of Health	Reports on the first births from Mexican mothers infected with COVID-19	Reports about health professionals’ influence on the discontinuation of breastfeeding among mothers

**Table 2:** SWOT operational definitions and examples of classification of information based on this analysis

Category	Operational definition*	Example
Strengths	Current actions, messages, policies, or programs that enable breastfeeding	The Mexican Ministry of Health promoting breastfeeding continuation during the COVID-19 pandemic
Weaknesses	Actions, messages, policies, or programs currently in place that negatively affect breastfeeding	Newborns separated from their mothers during the birth hospitalization due to fear of virus transmission from mothers to newborns.
Opportunities	Actions, messages, policies, or programs not currently in place that may enable breastfeeding	United Nations Children’s Fund (UNICEF) Mexico and the Mexican government initiating a new Cooperation Program for the 2020–2025 period.
Threats	Actions, messages, policies or programs not currently in place that may negatively affect breastfeeding	The COVID-19 pandemic projected to have collateral effects on health, not directly associated to the virus transmission, such as reduction in breastfeeding rates, delay in diagnoses, and mental health issues

* Adapted from Ferré-Eguiluz et al (42)

**Table 3:** Mentions classified by type of media

Type of media	Mentions, n (%)
Internet	848 (83.6%)
Newspapers	105 (10.4%)
TV	36 (3.6%)
Magazines	17 (1.7%)
Radio	8 (0.8%)

**Table 4:** Positive information found about breastfeeding and COVID-19

Theme	Mentions, n (% of total)
Recommendations to keep breastfeeding during the pandemic	90 (8.9%)
Reports of no evidence that COVID-19 could be transmitted via breastmilk	51 (5%)
Announcement that the Mexican Ministry of Health would promote breastfeeding during social distancing and lockdown measures	29 (2.9%)
Feeding recommendations during the pandemic made from United Nations (UN) agencies to the Mexican government	12 (1.2%)

**Table 5:** Results from the SWOT* analysis

**Strengths**	Follow-up of the World Breastfeeding Week Recommendations to keep breastfeeding during the pandemic Mexican Ministry of Health’s efforts to promote breastfeeding during the pandemic Comments about the importance of breastfeeding from Mexican Ministry of Health’s and UNICEF’s experts
**Weaknesses**	Lack of female representation in decision-making at the policy level and in breastfeeding events Lack of meaningful public policies to protect breastfeeding Separation of mother-infant dyads during the birth hospitalization period during the COVID-19 pandemic
**Opportunities**	UN agencies’ general nutrition recommendations during the pandemic, and breastfeeding highlighted as a measure to combat food vulnerability and insecurity New Cooperation Program between UNICEF Mexico and the Mexican government for the 2020–2025 period, where breastfeeding protection and promotion can be incorporated Non-governmental organizations’ demands to regulate breast milk substitute donations during the pandemic promoted by pharmacies and BMS companies to the general public
**Threats**	Increase of marketing of breast milk substitutes during the pandemic Work inequities during lockdown measures (i.e. women taking a disproportionate role in caring for children) Increasing C-section rates, which are associated with reduced breastfeeding Influence of medical doctors on women’s decisions not to breastfeed

* SWOT refers to strengths, weakness, opportunities, and threats

## List Of Abbreviations

AMAI: Mexican Association of Market and Opinion Intelligence Agencies (for its acronym in Spanish)
BMS: Breastmilk substitutes
ENCOVID-19: Survey on COVID-19 Effects on Wellness in Mexican Households (for its acronym in Spanish)
GILPALM: Permanent Interinstitutional Support Group for Breastfeeding (for its acronym in Spanish)
NGO: Non-governmental organization
SARS-CoV-2: Severe acute respiratory syndrome coronavirus 2
SWOT: Strengths, weaknesses, opportunities, threats
UN: United Nations
UNICEF: United Nations Children’s Fund
UK: United Kingdom
USA: United States of America
WHO: World Health Organization
